# Characterization of large and small-plaque variants in the Zika virus clinical isolate ZIKV/Hu/S36/Chiba/2016

**DOI:** 10.1038/s41598-017-16475-2

**Published:** 2017-11-23

**Authors:** Fumihiro Kato, Shigeru Tajima, Eri Nakayama, Yasuhiro Kawai, Satoshi Taniguchi, Kenichi Shibasaki, Masakatsu Taira, Takahiro Maeki, Chang Kweng Lim, Tomohiko Takasaki, Masayuki Saijo

**Affiliations:** 1 Department of Virology I, National Institute of Infectious Diseases, 1-23-1 Toyama, Shinjuku, Tokyo, 162-8640 Japan; 2 Division of Biosafety Control and Research, National Institute of Infectious Diseases, 1-23-1 Toyama, Shinjuku, Tokyo, 162-8640 Japan; 3 Division of Virology, Chiba Prefectural Institute of Public Health, 666-2 Nitona, Chuo, Chiba, 270-1422 Japan; 4 Kanagawa Prefectural Institute of Public Health, 1-3-1 Shimomachiya, Chigasaki, Kanagawa 253-0087 Japan

## Abstract

An Asian/American lineage Zika virus (ZIKV) strain ZIKV/Hu/S36/Chiba/2016 formed 2 types in plaque size, large and small. Genomic analysis of the plaque-forming clones obtained from the isolate indicated that the clones forming small plaques commonly had an adenine nucleotide at position 796 (230^Gln^ in the amino acid sequence), while clones forming large plaques had a guanine nucleotide (230^Arg^) at the same position, suggesting that this position was associated with the difference in plaque size. Growth kinetics of a large-plaque clone was faster than that of a small-plaque clone in Vero cells. Recombinant ZIKV G796A/rZIKV-MR766, which carries a missense G796A mutation, was produced using an infectious molecular clone of the ZIKV MR766 strain rZIKV-MR766/pMW119-CMVP. The plaque size of the G796A mutant was significantly smaller than that of the parental strain. The G796A mutation clearly reduced the growth rate of the parental virus in Vero cells. Furthermore, the G796A mutation also decreased the virulence of the MR766 strain in IFNAR1 knockout mice. These results indicate that the amino acid variation at position 230 in the viral polyprotein, which is located in the M protein sequence, is a molecular determinant for plaque morphology, growth property, and virulence in mice of ZIKV.

## Introduction

Zika virus (ZIKV) in the genus *Flavivirus* and the family *Flaviviridae* was first isolated from a sentinel rhesus monkey in Uganda and from mosquito in 1947 and 1948, respectively^1,2^. ZIKV infection in humans was first identified in Uganda and the United Republic of Tanzania in 1952. ZIKV was detected in several countries, not only in Africa but also in Asia. However, fewer than 20 cases of ZIKV infection in humans were confirmed in the 20th century^2^. The first outbreak of ZIKV infection in humans was identified in Yap Island in the Federal State of Micronesia in 2007; around 70% of residents over 3 years of age were infected with ZIKV, and around 18% of people infected with ZIKV developed clinical symptoms^2,3^. Sporadic ZIKV infections also occurred in Southeast Asia since the outbreak^4–8^. The outbreak of ZIKV infections was also confirmed in French Polynesia in the South Pacific, with an estimated number of ZIKV infection cases of approximately 30,000^9^. ZIKV infection epidemics spread to the other Pacific regions, as well as the Americas in 2014–2015. In 2016, patients with ZIKV disease were reported in several countries in Southeast Asia, including Singapore, Thailand, Vietnam, and the Philippines^10^. ZIKV is transmitted to humans mainly through the bite of *Aedes* mosquitoes^11^. Non-vector transmission of ZIKV was also reported to occur through transfusion, transplantation, and sexual intercourse^12,13^. The clinical symptoms caused by ZIKV infection are generally mild. Common manifestations are fever, rash, headache, joint and muscle pain, and conjunctivitis. A rash was observed in over 90% of patients with symptomatic ZIKV infections, whereas 60–70% of patients developed a fever. When pregnant women are infected with ZIKV, the fetus can be infected with ZIKV through the placenta, causing congenital ZIKV infections in fetus with the following symptoms: microcephaly, sensorineural abnormalities, cerebral calcifications, and abortion^14,15^. ZIKV infection also caused Guillain–Barré Syndrome (GBS) in some patients during the outbreak in French Polynesia in 2013–2014^16,17^. The World Health Organization (WHO) declared ZIKV epidemics in the Pacific countries and territories and the Americas, and its associated complications a public health emergency of international concern (PHEIC) on February 1st, 2016 though the PHEIC was lifted in November 2016.

ZIKV genome is composed of a single-stranded, positive-sense RNA, which encodes 3 structural proteins (C, prM, and E) and 7 nonstructural proteins (NS1, NS2A, NS2B, NS3, NS4A, NS4B, and NS5) in one open reading frame. ZIKV is genetically divided into 2 lineages, African and Asian/American lineages. The recent epidemics in Pacific regions and the Americas were caused by the introduction of Asian/American lineage ZIKV^18,19^.

Reverse genetics system is a powerful tool for analysis of the genetic determinants in replication and pathogenesis of flaviviruses and for understanding the nature of virus isolates. Infectious clones of ZIKV were constructed with the reverse genetics system^20–26^. Mouse models of ZIKV infection were established using engineered mice such as interferon receptor knockout (KO) mice, interferon regulatory factor-KO mice, STAT2-KO mice, and immune competent SJL and Balb/c strains^27–37^. These are valuable models not only for studying the pathogenesis of ZIKV but also for evaluating vaccines and drugs against ZIKV infection.

Twelve imported cases of ZIKV infections have been reported in 2016 in Japan. Recently, we isolated a ZIKV strain (ZIKV/Hu/S36/Chiba/2016) from serum of a patient, who returned from Fiji to Japan in April 2016^38^. Phylogenetic analysis indicated that the isolate belonged to Asian/American lineage ZIKV and showed the highest homology to the Tonga strain isolated in 2016^38^. During the characterization process of the ZIKV/Hu/S36/Chiba/2016 isolate *in vitro*, it was revealed that the isolate included at least two variants with different phenotypes. The characteristics of these two variants in terms of plaque formation, growth capacity *in vitro*, and virulence were elucidated. In this study, we established a reverse genetics system for ZIKV using the MR766 strain^39^. The genetic determinant that caused the difference in phenotypes was determined using the reverse genetics system.

## Results

### ZIKV/Hu/S36/Chiba/2016 isolate contains two types of ZIKV

**Figure 1:**
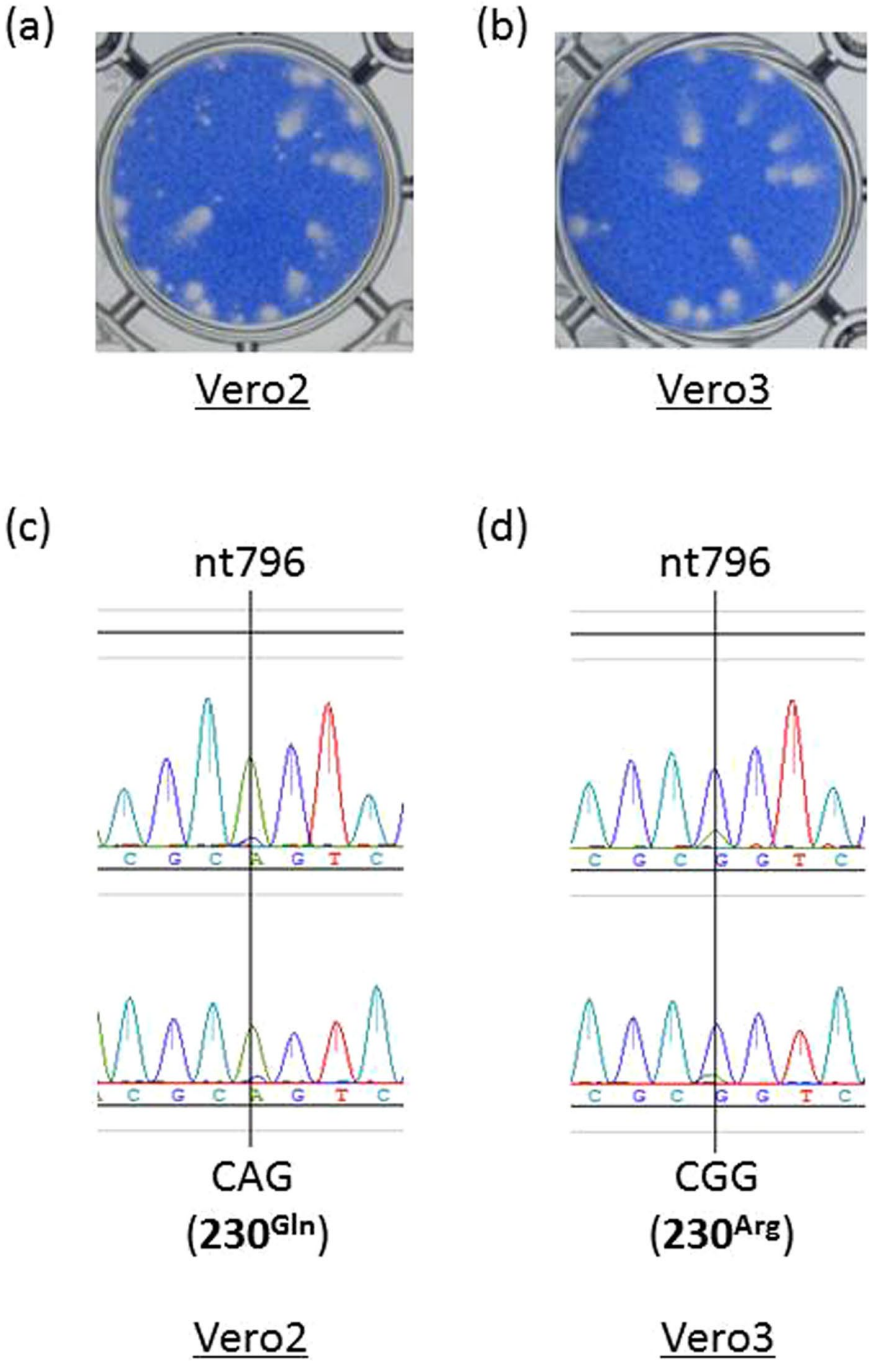
Plaque phenotypes of the ZIKV/Hu/S36/Chiba/2016 isolate in Vero cells (**a**,**b**). Plaque morphology of ZIKV/Hu/S36/Chiba/2016-Vero2 (**a**) and ZIKV/Hu/S36/Chiba/2016-Vero3 (**b**) in Vero cells cultured with the supernatants amplified two and three times, respectively (c and d). Sequencing electropherograms at nucleotide position 796 in ZIKV/Hu/S36/Chiba/2016-Vero2 (**c**) and -Vero3 (**d**). Two different primers (upper and lower electropherograms) were used for the sequencing of the region.

The virus was isolated by adding diluted serum of the patient to the culture medium of Vero cells and subsequent blind passage of the culture supernatant to Vero cells. The ZIKV/Hu/S36/Chiba/2016 isolate at this stage was defined as “ZIKV/Hu/S36/Chiba/2016-Vero2”, and it formed morphologically different types of plaques, which were large and small in size, in Vero cells (Fig. 1a). The ratio of the number of the large to small plaques was approximately 1:1. When a small aliquot of the ZIKV/Hu/S36/Chiba/2016-Vero2 isolate was amplified in Vero cells, the amplified virus was defined as “ZIKV/Hu/S36/Chiba/2016-Vero3”. Most of the plaques formed by ZIKV/Hu/S36/Chiba/2016-Vero3 were large in size (Fig. 1b). The complete nucleotide sequence of the ZIKV/Hu/S36/Chiba/2016-Vero2 and -Vero3 isolates revealed that there was only one nucleotide difference between these viruses at position 796, which corresponds to position 230 in the viral polyprotein and is located in the structural M protein coding region. The major and minor nucleotides at position 796 in ZIKV/Hu/S36/Chiba/2016-Vero2 were adenine and guanine, respectively (Fig. 1c). By contrast, the major and minor nucleotides at the same position were guanine and adenine in ZIKV/Hu/S36/Chiba/2016-Vero3, respectively (Fig. 1d). Moreover, the adenine-to-guanine nucleotide change at 796 (A796G) led to an amino acid change at position 230, Gln to Arg (Gln230Arg). To clarify whether the sequence difference caused the alteration in plaque morphology, ZIKV clones were obtained from the ZIKV/Hu/S36/Chiba/2016-Vero2 solution using a limiting dilution method. Nineteen clones were obtained. The complete nucleotide sequences of 10 out of 19 clones were determined (Table 1). Several nucleotide variations were seen among the clones. All 5 clones forming small plaques had an adenine nucleotide at position 796, but all 4 clones forming large plaques had a guanine at the same position. One clone (#28), which was a mixed population of large-plaque and small-plaque viruses, had a mix of adenine and guanine at that position. These results suggest that the amino acid at position 230 was associated with the difference in plaque formation.

**Table 1:** Plaque size and nucleotide at position 796 of the virus clones isolated from the parent strain ZIKV/Hu/Chiba/S36/2016.

Clone #	Plaque size	Nucleotide at position 796	Amino acid at position 230
3	Large	G	Arg
5	Small	A	Gln
6	Large	G	Arg
8	Small	A	Gln
28	Mix	G and A	Arg/Gln
53	Small	A	Gln
61	Small	A	Gln
68	Large	G	Arg
69	Large	G	Arg
70	Small	A	Gln

### ***In vitro*** growth of the large-plaque and small-plaque variants of ZIKV

**Figure 2:**
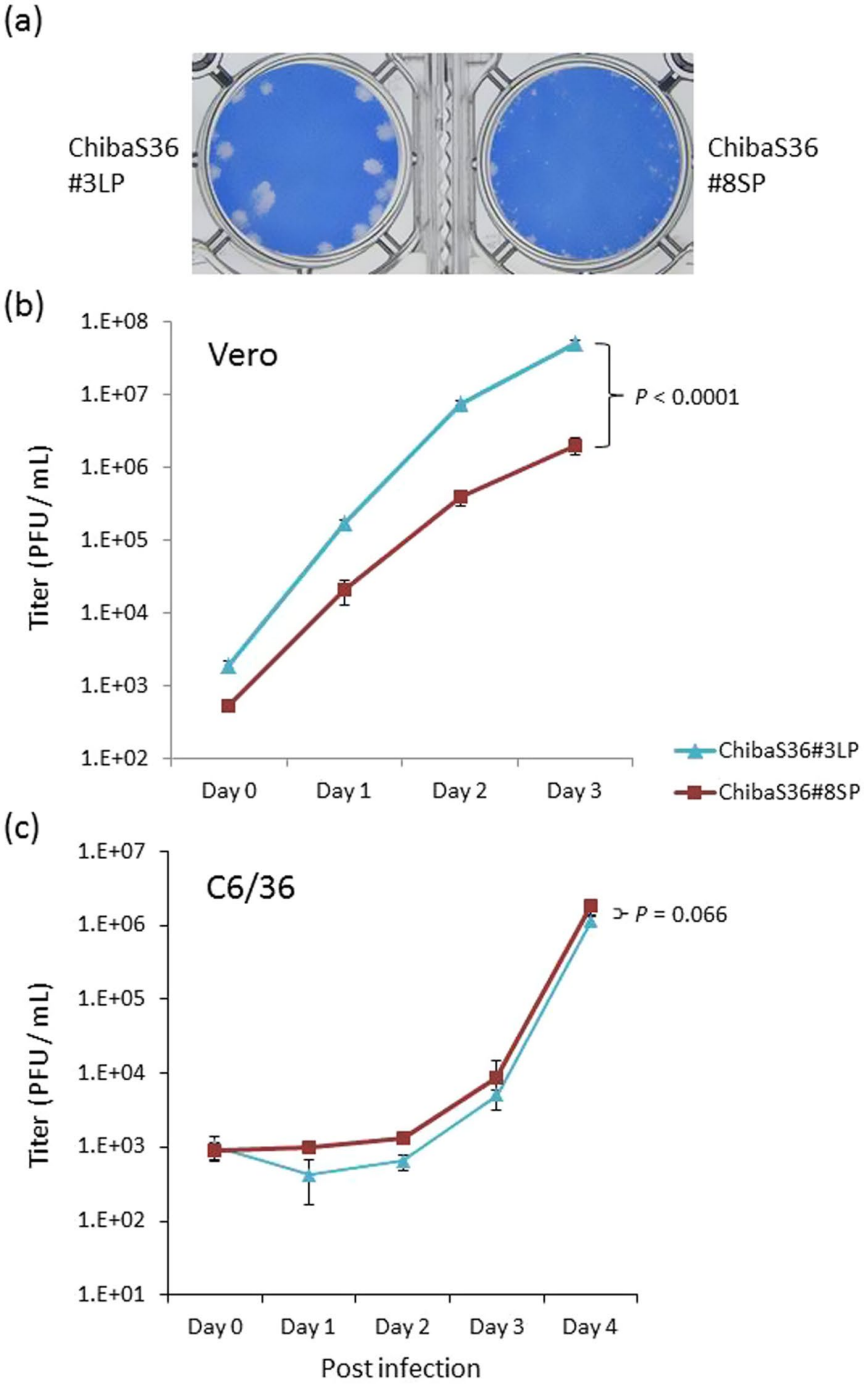
Growth properties of the large-plaque ChibaS36#3LP and small-plaque ChibaS36#8SP clones of ZIKV/Hu/S36/Chiba/2016. (**a**) Plaque morphology of the clones in Vero cells. (**b**,**c**) Growth kinetics of each clone in Vero cells (**b**) and in C6/36 cells (**c**). Cells were plated into 6-well culture plates and infected with the ZIKV clones at a multiplicity of infection of 0.01 PFU/cell. Values represent the mean and standard deviation from 3 independent experiments. *P* values were calculated by using two-way ANOVA test.

To examine the growth properties of large and small-plaque ZIKV, two clones, designated as ChibaS36#3LP (large plaque) and ChibaS36#8SP (small plaque), were selected from the 19 clones (Fig. 2a). On the one hand, ChibaS36#3LP grew faster than ChibaS36#8SP in Vero cells (Fig. 2b). Infectious titer in the culture supernatant reached 5 × 10^7^ PFU/mL at 72 hours after inoculation, and ChibaS36#3LP titer was over 20-times higher than that of ChibaS36#8SP. On the other hand, both clones showed slower replication kinetics in mosquito C6/36 cells than in Vero cells, and there was less difference in growth rate between the clones (Fig. 2c).

### Generation of a recombinant ZIKV G796A/rZIKV-MR766

**Figure 3:**
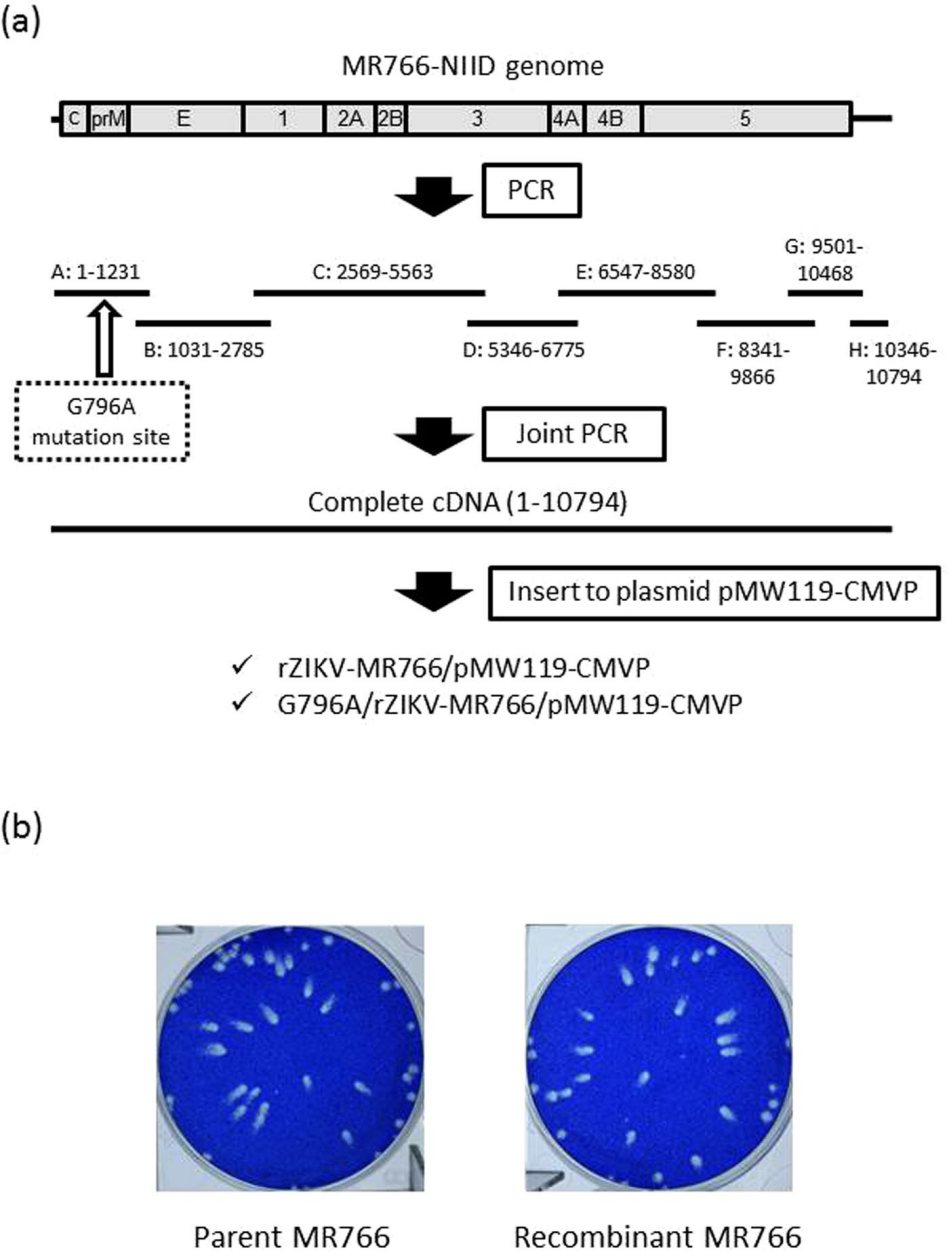
(**a**) Schematic representation of the construction of ZIKV infectious molecular clone rZIKV-MR766/pMW119-CMVP and mutant clone G796A/rZIKV-MR766/pMW119-CMVP. (**a**–**h**) RT-PCR fragments and nucleotide regions on the MR766 genome. A detailed explanation of the strategy is described in the Methods section. (**b**) Plaque morphology of the recombinant rZIKV-MR766 virus and its parental strain MR766 in Vero cells.

To demonstrate whether the amino acid difference at position 230 was associated with plaque formation and growth capacities in Vero cells, a recombinant ZIKV with or without the mutation at position 796 (G796A) was generated by using a reverse genetic system. A reverse genetics system for ZIKV was established by constructing an infectious molecular clone of the ZIKV MR766 strain rZIKV-MR766/pMW119-CMVP (Fig. 3a). Vero cells were transfected with the plasmid, and the culture supernatant fluid was recovered. The recombinant virus nucleotide sequence had no mutation, and the plaque size and the growth kinetics of the recombinant virus were identical to the parental virus, indicating that the reverse genetics system for producing infectious recombinant ZIKV was established (Figs 3b and S1).

ChibaS36#3LP and MR766 strain possessed a guanine at the position 796; therefore, a guanine-to-adenine substitution (G796A) was introduced at position 796 on the genomic region of the MR766 clone to construct a mutant clone G796A/rZIKV-MR766/pMW119-CMVP. The mutant recombinant virus G796A/rZIKV-MR766 was produced successfully by transfecting Vero cells with the mutant clone.

### Growth kinetics and plaque formation of recombinant ZIKV G796A/rZIKV-MR766 and parental MR766 virus

**Figure 4:**
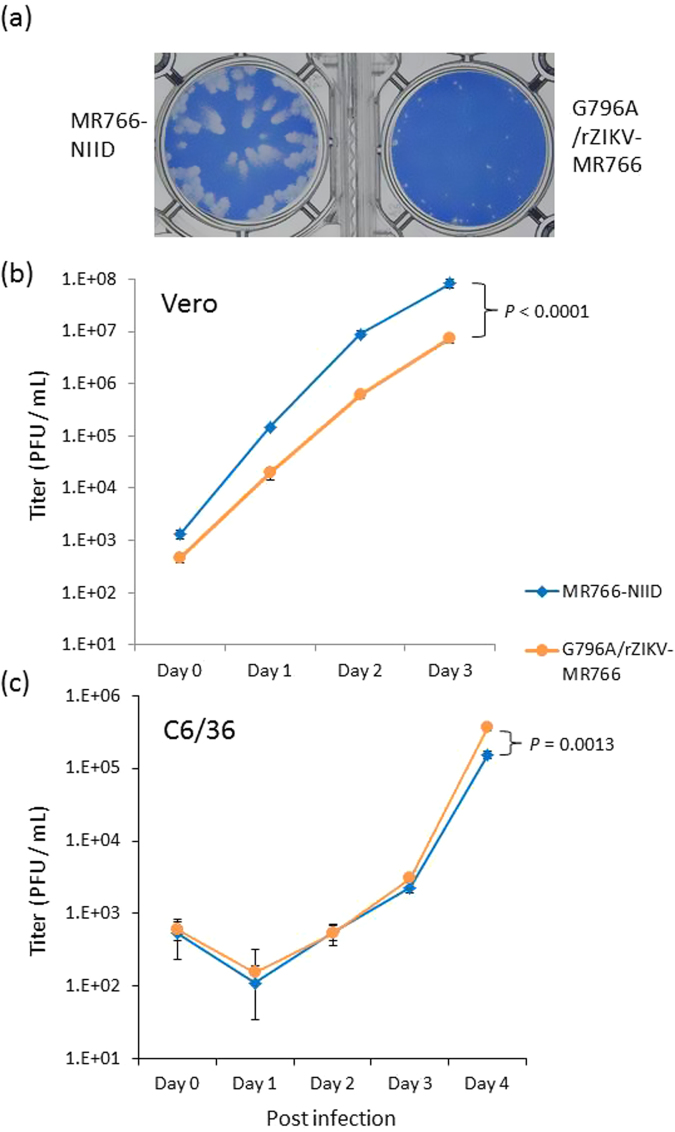
Growth properties of the recombinant G796A/rZIKV-MR766 virus. (**a**) Plaque morphology of the recombinant and parental MR766 strains in Vero cells. (b and **c**) Growth kinetics of the recombinant and parental viruses in Vero cells (**b**) and in C6/36 cells (**c**). Cells were plated into 6-well culture plates and infected with the viruses at a multiplicity of infection of 0.01 PFU/cell. Values represent the mean and standard deviation from three independent experiments. *P* values were calculated by using two-way ANOVA test.

The plaque size of G796A/rZIKV-MR766 was clearly smaller than that of the parental strain (Fig. 4a). Furthermore, the growth kinetics of G796A/rZIKV-MR766 was also significantly slower than that of the parental strain in Vero cells, indicating that the G796A mutation alone caused reductions in plaque size and growth properties in Vero cells (Fig. 4b). However, the parental and G796A mutant viruses grew similarly in C6/36 cells, although there was a slight difference between the two viruses at day 4 (Fig. 4c).

### Pathogenesis of G796A/rZIKV-MR766 and parental MR766 virus in IFNAR1-KO mice

**Figure 5:**
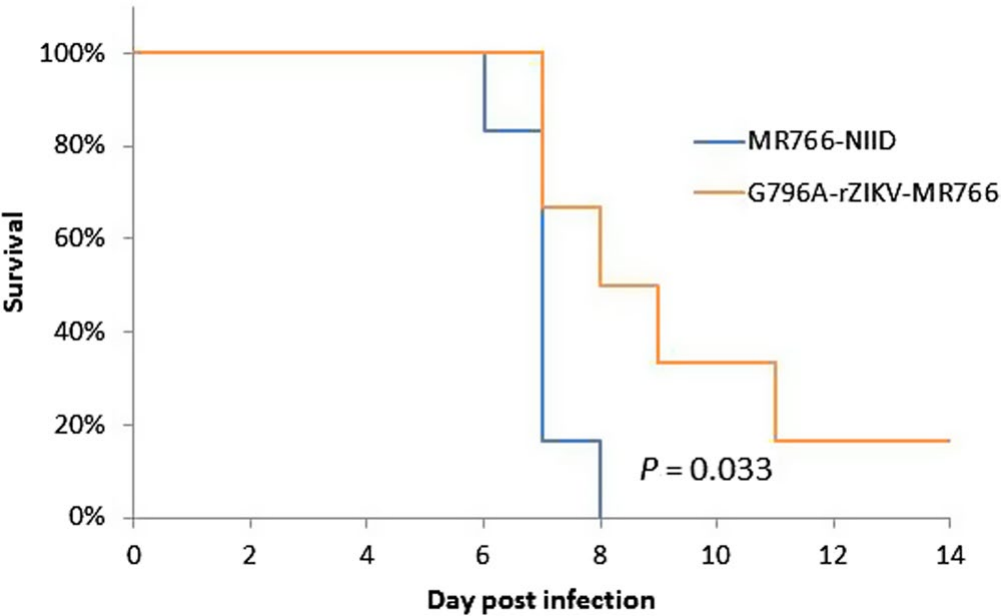
Survival rate of mice infected with 1 × 10^2^ PFU of MR766 or G796A/rZIKV-MR766. Mice in the two groups were monitored for 14 days after virus challenge.

Previous reports showed that interferon alpha/beta receptor 1 gene-knockout (IFNAR1-KO) mice were vulnerable to MR766 infection^30^. Using the mouse model, we evaluated the virulence of recombinant G796A/rZIKV-MR766 (Fig. 5). All the mice infected with 1 × 10^2^ PFU of the MR766 strain died within 8 days. However, the survival of mice infected with G796A/rZIKV-MR766 from inoculation was longer than that of mice infected with MR766, and 5 out of 6 mice inoculated with recombinant G796A/rZIKV-MR766 died within 11 days and one survived. Statistical analysis (log-rank test) indicated that there was a significant difference in the survival rates between the two groups (*P* = 0.033). Body weight loss of mice inoculated with G796A/rZIKV-MR766 was also smaller than that of mice inoculated with the parental MR766 virus (Figure S2). The viremia level in the mice inoculated with G796A/rZIKV-MR766 was lower than that in the MR766-inoculated mice (Figure S3).

### Nucleotide sequencing at position 796 of ZIKV/Hu/Chiba/S36/2016 from the patient serum and the virus isolated using C6/36 cells

To confirm whether the nucleotide at position 796 in ZIKV sequence was mainly adenine or guanine, the nucleotide sequence was directly determined from the serum sample (Table 2). Guanine was detected at that position in the serum sample, suggesting that the majority of ZIKV in the patient serum are large-plaque variants. We also amplified ZIKV/Hu/S36/Chiba/2016-Vero2, which was then propagated in C6/36 cells three times (ZIKV/Hu/S36/Chiba/2016-Vero2-C6/3); the nucleotide sequence of the virus was then determined (Table 2). Both adenine and guanine at position 796 were detected, suggesting that the adenine-type and guanine-type viruses coexisted in the culture supernatant fluid of C6/36 cells infected with ZIKV/Hu/S36/Chiba/2016-Vero2-C6/3.

**Table 2:** Nucleotide at position 796 of the isolates of ZIKV/Hu/S36/Chiba/2016.

Virus	Major nucleotide
Two passages in Vero cells (Vero2)	A
Three passages in Vero cells (Vero3)	G
Two passages in Vero cells and then Three passages in C6/36 cells (Vero2-C6.3)	A and G*
Serum from the patient	G

*A and G nucleotides were mixed in the culture supernatant fluid.

## Discussion

In this study, we demonstrated that the viral solution of the ZIKV/Hu/S36/Chiba/2016-Vero2 isolate contained at least two phenotypes of ZIKV with different plaque size formation and growth kinetics *in vitro* and that a nucleotide at position 796 (amino acid residue at position 230 in the viral polyprotein), which locates in M protein, was responsible for the differences. We also proved that the G796A mutation decreased the virulence of ZIKV MR766 strain in IFNAR1-KO mice.

Small-plaque variants were found in low-passaged viral solutions and gradually disappeared from the culture supernatant by repeated passages in Vero cells (Fig. 1). Therefore, it was speculated that these small-plaque variants represented the largest population in the patient serum. However, the sequencing data indicated that most viruses in the patient serum consisted of the large-plaque variant, because the nucleotide detected was mainly guanine at position 796. How the small-plaque variant was isolated by inoculating Vero cells with the serum samples, as well as the large-plaque variant, remains to be elucidated. Both the large and small-plaque variants were found after passaging the isolate (Vero2) in C6/36 cells (Table 2). The growth capacity of ZIKV/Hu/S36/Chiba/2016 in C6/36 cells was clearly lower than that in Vero cells, but no clear difference in growth rate was observed between the large and small-plaque clones (Fig. 2). These results suggest that the small-plaque variant may disappear more slowly in C6/36 cells than in Vero cells through multiple passages. The difference in growth kinetics between the parental MR766 strain and the G796A mutant virus was also small in C6/36 cells, indicating that the amino acid at position 230 is not important for the ZIKV growth in C6/36 cells.

**Figure 6:**
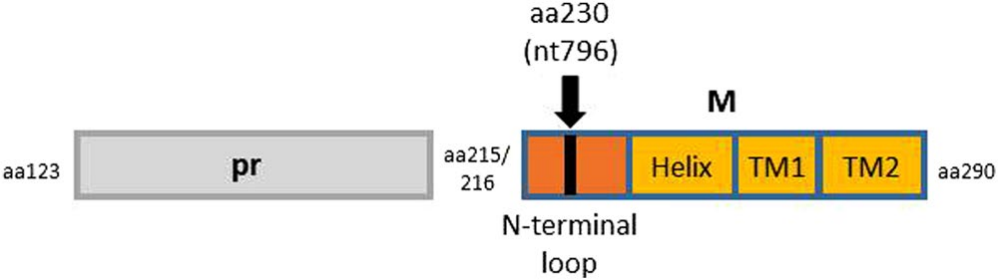
Schematic representation of prM protein of ZIKV. Numbers indicate amino acid positions on the polyprotein. TM, transmembrane.

In flaviviruses, prM protein is a membrane glycoprotein and forms a heterodimer with envelope E protein on the surface of virus particles. The roles of prM protein are to assist in the proper folding of E protein and to prevent E protein from undergoing acid-catalyzed rearrangement to the fusogenic form during the secretory pathway. In the maturation process of virion, prM is cleaved into pr and M regions by a cellular protease furin. M region of ZIKV consists of 75 amino acid residues (about 10 kDa) and an N-terminal loop, a membrane-associated helix, and two transmembrane (TM) domains (Fig. 6)^40^. The amino acid at position 230 is located on the loop region. The TM and helix domains are deemed to be involved in assembly and maturation of virus particles and dimerization with E protein^41–43^. However, there is little information about the N-terminal loop. In this study, we showed that the amino acid substitution in the loop influences ZIKV growth *in vitro* and its virulence *in vivo*. This raises the possibility that the region plays an important role in ZIKV replication *in vitro* and *in vivo* in mammalian cells. The N-terminal loop is considered to lie within the hole in between the homodimers of E ectodomain^44,45^, suggesting that the loop may also be associated with the formation of ZIKV particles. Recent report demonstrated that a single mutation in the ZIKV prM causes fetal microcephaly in mice, suggesting that ZIKV prM is involved in the pathogenicity of ZIKV^46^.

The African lineage MR766 strain was passaged in mouse brain repeatedly and has adapted to mice, in which it was detected in various tissues, such as blood, spleen, liver, kidney, brain, spinal cord, placenta, testis, and body fluids, and led to dysfunction of placenta and testis, death and reduced proliferation of neural progenitor cells, and fetal brain infection in the animal models^47^. Our results of *in vitro* analyses suggest that the G796A mutation influenced ZIKV growth in IFNAR1-KO mice and *in vivo*, the growth rate of the mutant G796A/rZIKV-MR766 virus in the mice was lower than that of the parental MR766 virus.

We established the reverse genetics of ZIKV using the MR766 strain in the present study. Using this system, we could demonstrate clearly that adenine at position 796 in the ZIKV genome was involved in small-plaque formation and lowered growth capacity *in vitro* and *in vivo*. Thus, the reverse genetics system that we developed enables us to understand of the nature of ZIKV in more details.

## Methods

### Viruses, cell culture, plaque assay, and virus cloning

The ZIKV strain ZIKV/Hu/S36/Chiba/2016 strain^38^ and the MR766 strain (MR766-NIID, accession no. LC002520), originally isolated from a rhesus monkey in the Zika forest of Uganda in 1947 and maintained in the National Institute of Infectious Diseases (NIID), Japan, were used in this study. These viruses were propagated in Vero cells (strain 9013) and mosquito-derived C6/36 cells. Vero cells and C6/36 cells were cultured at 37 °C and 28 °C, respectively, in 5% CO_2_ in Eagle’s minimum essential medium (MEM) supplemented with 10% heat-inactivated fetal bovine serum (FBS) and 100 U/mL penicillin–streptomycin. To determine the infectious titer of the virus, a plaque assay was performed as described previously^48^. Briefly, Vero cells (2 × 10^5^ cells/well) were plated in 12-well plates and inoculated with serially diluted viruses. Four (Fig. 3) or five (Figs 1, 2 and 4) days after inoculation, the cells were fixed with a 3.7% (v/v) formaldehyde solution in phosphate-buffered saline and then were stained with a methylene blue solution for 2 hours.

For cloning viruses, the ZIKV/Hu/S36/Chiba/2016-Vero2 solution, which was obtained by inoculating the patient serum into Vero cells and subsequent passage of the supernatant in Vero cells^38^, was diluted with MEM supplemented with 2% FBS (MEM-2% FBS) and then inoculated into Vero cells in 24-well plate at 0.5–1.0 infectious virus particle/well. Seven days after inoculation, the supernatants were recovered and then used to check the plaque size of the clones using a plaque assay as described above. Ten out of the 19 clones obtained were used for determination of the complete nucleotide sequence.

### Genome sequencing

Viral RNA was isolated from culture supernatants and serum of the Zika patient^38^ using High Pure Viral RNA Kit (Roche Diagnostics, IN, USA) and was used for synthesis and amplification of cDNA using a one-step RT-PCR method using PrimeScript II High Fidelity One Step RT-PCR kit (TAKARA Corp., Shiga, Japan). Viral cDNA were amplified by PCR using the primer sets described in Supplementary Table 1. PCR products were sequenced using the dideoxy sequencing reaction kit (BigDye terminator method; Thermo Fisher Scientific, Waltham, MA, USA) and Genetic Analyzer 3500 (Thermo Fisher Scientific) with primers specific for ZIKV. The raw sequencing data were assembled to reconstruct the complete ZIKV genome and translated into amino acid sequences using GENETYX gene analysis software (Genetyx Corp., Tokyo, Japan).

### Production of recombinant ZIKV

A plasmid pMW119-CMVP, which contains a CMV promoter at 5′, and ribozyme and polyA tail sequence at 3′ of the viral sequence^49^, was amplified with the primers pCMV.del.f and pCMV.del.r (Supplementary Table 1) and digested with the restriction enzyme Dpn I. MR766-NIID RNA was extracted and used for the synthesis of viral cDNA using ReverTra Ace (TOYOBO, Osaka, Japan). Eight regions (A to H regions), which included the overlapped sequences adjacent to the other regions, were amplified by PCR using Q5 Hot Start High-Fidelity Master Mix (New England Biolabs, Ipswich, MA, USA) and the primers (Supplementary Table 1 and Fig. 3a). The fragments were mixed and then amplified by PCR using 5′-terminal and 3′-terminal primers, pCMVIF.ZKV001f, and pCMVIF.ZKV10794r (Supplementary Table 1), to synthesize the complete MR766-NIID cDNA. The full-length cDNA and the amplified pMW119-CMVP were ligated using In-Fusion HD (TaKaRa-Clontech, Shiga, Japan). Complete plasmids were amplified in NEB Stable Competent cells (New England Biolabs) and then recovered. Vero cells were transfected with the recombinant clone rZIKV-MR766/pMW119-CMVP using X-tremeGene HP (Roche) transfection reagent. After incubation of the transfected cells for 4 days, the culture supernatant was collected and inoculated onto Vero cells. Four days after inoculation, the culture supernatant was recovered, and its virus titer was measured with a plaque assay. A recombinant molecular clone, G796A/rZIKV-MR766/pMW119-CMVP, was constructed using the ZIKV strain MR766-NIID backbone. A guanine nucleotide at position 796 of rZIKV-MR766/pMW119-CMVP was replaced with an adenine nucleotide by site-directed mutagenesis (inverse PCR method) using primers RtoQ.f and RtoQ.r (Supplementary Table 1). The recombinant virus was recovered by transfection of Vero cells with the recombinant clone plasmid described above. Complete nucleotide sequence of the recombinant virus was determined, and it was confirmed that no spontaneous mutations were present on the genomes.

### Growth kinetics analysis

Cells were plated in a 6-well culture plate (3 × 10^5^ cells/well for Vero and 6 × 10^5^ cells/well for C6/36 cells) and infected with ZIKV, including recombinant ZIKV, at a multiplicity of infection of 0.01 plaque-forming units (PFU) per cell. Small aliquots of the media were recovered periodically, and the titer of the aliquots was determined by a plaque assay on Vero cells grown in 12-well culture plates. To evaluate the plaque size, Vero cells (3 × 10^5^ cells/well) were plated in 12-well plates and inoculated with the viruses. Five days after inoculation, cells were fixed and then stained with a methylene blue solution as described above. Statistical comparison of growth curves was performed using Prism (GraphPad Software, La Jolla, CA, USA) that uses the two-way ANOVA test.

### Mouse challenge

Mouse experiments were performed in accordance with the “Guidelines for animal experiments performed at National Institute of Infectious Diseases” under the approval (no. 116067) from the Animal Welfare and Animal Care Committee of the National Institute of Infectious Diseases, Japan. IFNAR1-KO C57BL/6 mice were provided by the RIKEN Bio Resource Center, Japan, through the National Bio-Resource Project of the MEXT, Japan^50,51^. All mice were bred and maintained in a specific-pathogen-free environment. Mice (7–9 weeks old, n = 6) were inoculated with 1 × 10^2^ PFU of each virus solution diluted with MEM-2% FBS through the subcutaneous route in the footpad. The mice were observed clinically, and their body weight was measured every day for 2 weeks after inoculation. Survival curve comparisons were performed using JMP13 software (SAS Institute Japan, Tokyo, Japan) statistical analysis with a log-rank test. Blood was collected after inoculation of the viruses at an interval of 2 days (3 mice, day 1, 3, and 5; the rest 3 mice, day 2, 4, and 6), and TCID50 was calculated as described previously^52^. The dataset are available from the corresponding author on reasonable request.

## Electronic supplementary material

Supplementary Information
