# Supplementary material for: Characterization of large and small-plaque variants in the Zika virus clinical isolate ZIKV/Hu/S36/Chiba/2016
Source: Sci Rep. 2017 Nov 23;7:16160. doi: 10.1038/s41598-017-16475-2 (PMC5701032; doi:10.1038/s41598-017-16475-2)
Supplement: Supplementary file 1 — Supplementary Information [file 41598_2017_16475_MOESM1_ESM.pdf]

Supplemental information

Characterization of large and small-plaque variants in the Zika virus clinical isolate ZIKV/Hu/S36/Chiba/2016

Fumihiro Kato[1], Shigeru Tajima[1]*, Eri Nakayama[1], Yasuhiro Kawai[2], Satoshi Taniguchi[1], Kenichi Shibasaki[1], Masakatsu Taira[3], Takahiro Maeki[1], Chang Kweng Lim[1], Tomohiko Takasaki[4], and Masayuki Saijo[1]

[1]Department of Virology I, National Institute of Infectious Diseases
[2]Division of Biosafety Control and Research, National Institute of Infectious
[3]Division of Virology, Chiba Prefectural Institute of Public Health
[4]Kanagawa Prefectural Institute of Public Health

*Corresponding author

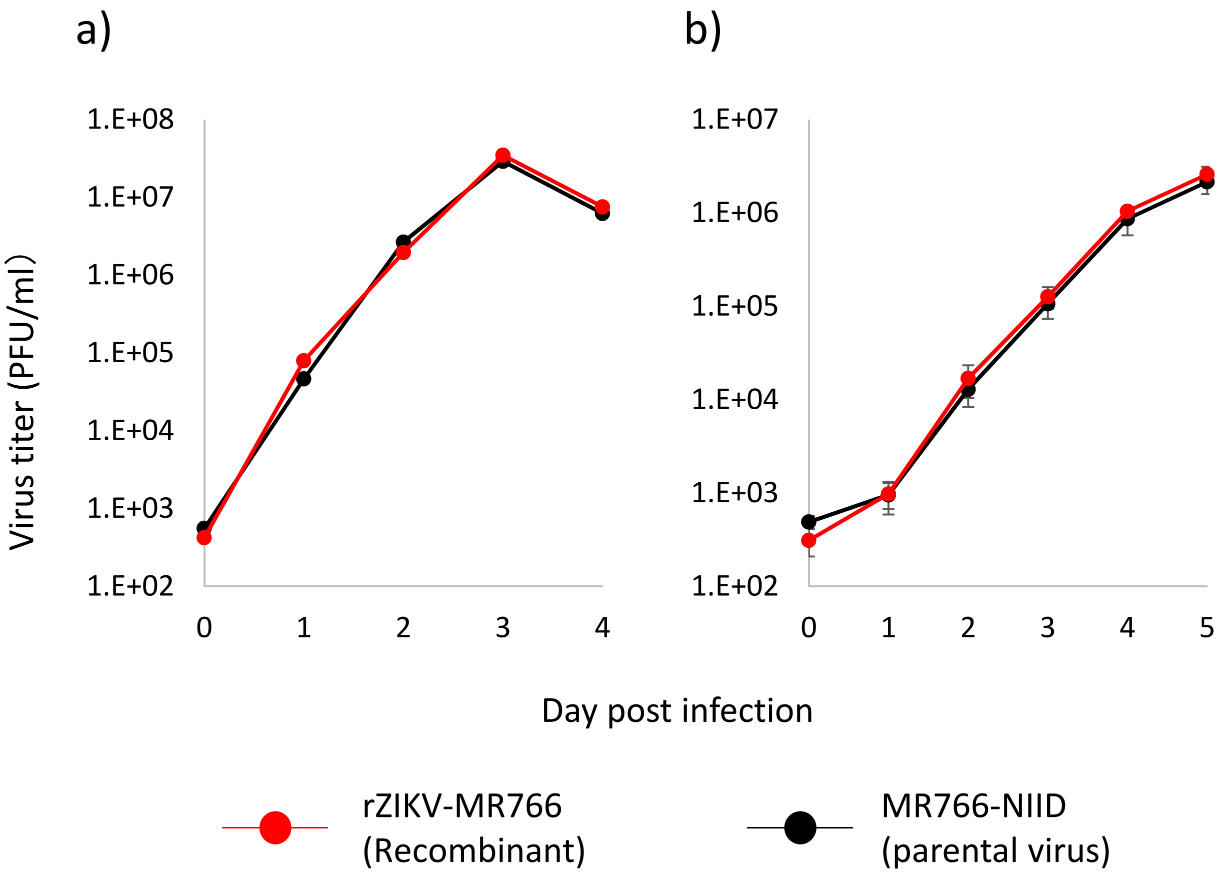

Figure S1. Growth kinetics of the recombinant rZIKV-MR766 and parental MR766 strains in Vero cells (a) and in C6/36 cells (b). Values represent the mean and standard deviation from three independent experiments.

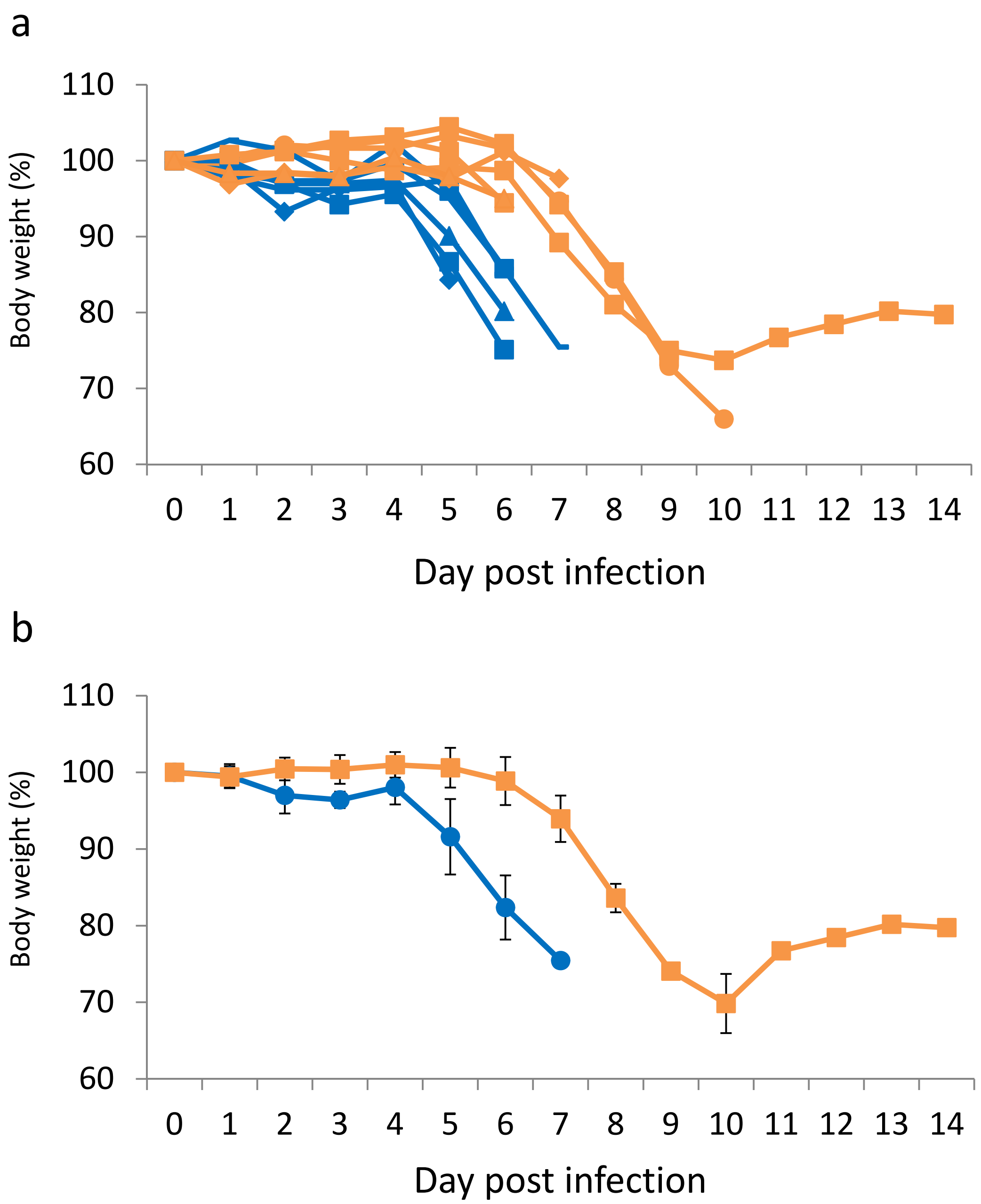

Figure S2. (a) Body weight of the IFNAR1-KO mice inoculated with the recombinant G796A mutant (orange lines, 6 mice) or parental MR766 (blue lines, 6 mice) ZIKV. **(b)** Mean body weight of the two mouse groups. Values also represent the standard deviation in the living mice.

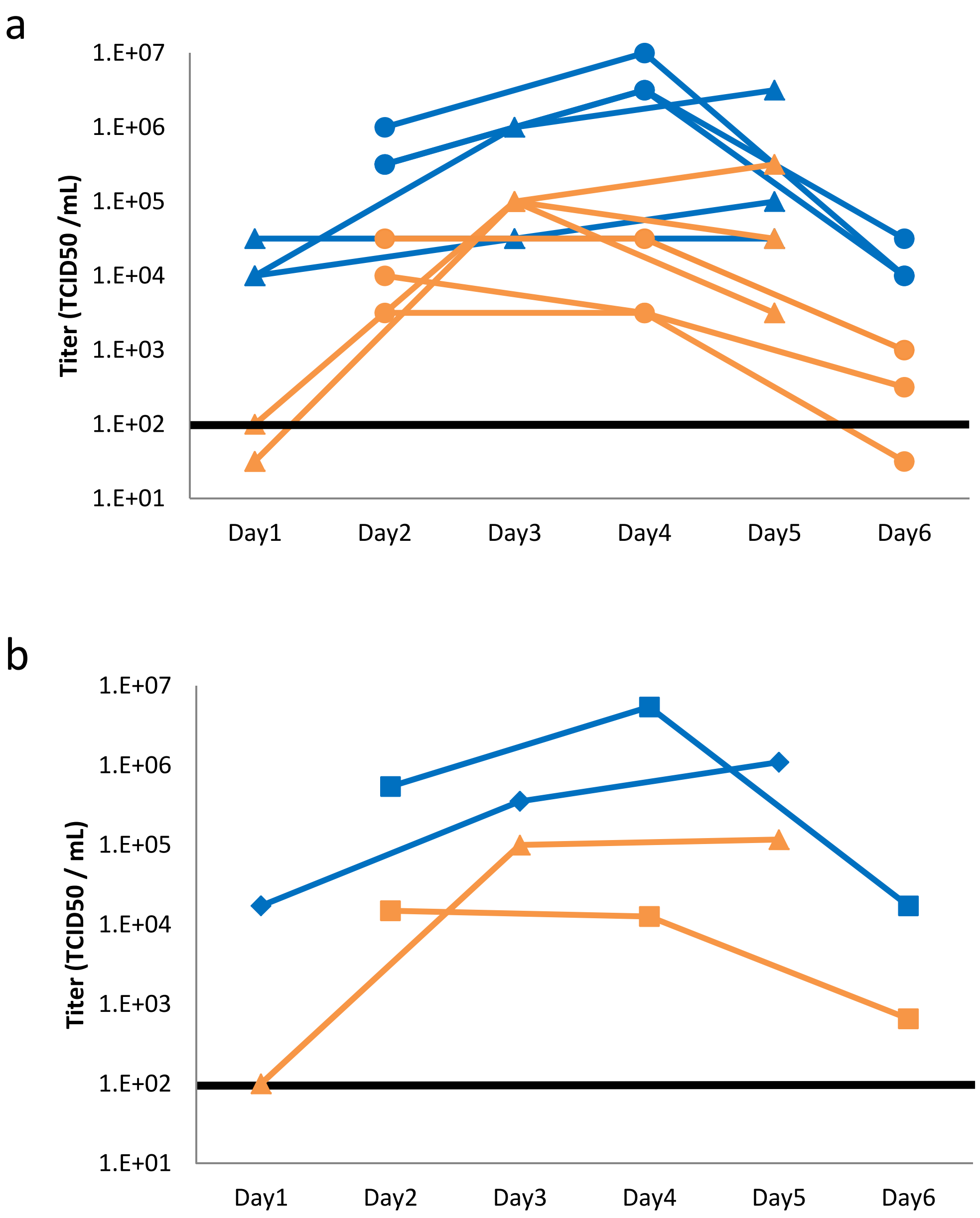

Figure S3. (a) Viremia levels in the IFNAR1-KO mice inoculated with the recombinant G796A mutant (orange lines, 6 mice) or parental MR766 (blue lines, 6 mice) ZIKV. Blood was collected on day 1, 3, and 5 (triangles, 3 mice) and on day 2, 4, and 6 (circles, 3 mice) after inoculation of the viruses, and TCID50 was calculated as described previously (Gardner et al. *Journal of Virology 84*: 8021-832, 2010). The horizontal bold line indicates the limit of detection (100 TCID50/mL). **(b)** Mean titer of the 4 mouse groups.

Table S1. Primers used for amplification of ZIKV genome

Region	Primer	Sequence (5'-3')
A	ZKV.001f	AGT TGT TGA TCT GTG TGA GTC
	ZKV.1231r	TTG CTT GTC AAG GTA GGC TTC
B	ZKV.1041f	ATG TTG TCT TGG AAC ATG GAG
	ZKV.2785r	TCT TTG TGG ACC TCT CCA CAT
C	ZKV.2569f	GTA CAA GTA CCA TCC TGA CTC
	ZKV.5563r	TCC TGG TGG TGT GGC AGT CAT
D	ZKV.5346f	ACG TCA CCC ATT CTG GGA CAG
	ZKV.6775r	GAC ACA TGC AAT TCT GGC TGG
E	ZKV.6547f	GCG AGC AGA GAC TGG AAG CAG
	ZKV.8580r	CTC CCA TGG TAG GCC CAT GTC
F	ZKV.8341f	CAT AAA AAG TGT GTC CAC CAC
	ZKV.9866r	CAA TCA GTT CAT CTT GGT GGC
G	ZKV.9501.f	ACA CAT TCA CCA ACT TGG TG
	ZKV.10468r	CTA TGA GCT TGG TTT CCC AGC T
H	ZKV.10346f	TCC ACA CCC GGA GTG TTG TAA
	ZKV.10794r2	AGA AAC CAT GGA TTT CCC CAC ACC GGC
Full genome	pCMVIF.ZKV001.f	TGG TTT AGT GAA CCG AGT TGT TGA TCT GTG TGA GT
	pCMVIF.ZKV10794.r	ATG CCA TGC CGA CCC AGA AAC CAT GGA TTT CCC CA
Vector	pCMV.del.f	GGG TCG GCA TGG CAT CTC CAC C
	pCMV.del.r	CGG TTC ACT AAA CCA GCT CTG
Inverse PCR	RtoQ.f	AAC CAG GTC TGC GAC TGC GTT TGC AAC TTC CTT GT
	RtoQ.r	GAT GTT CCT TCA ACG TTT GCG T
